# Pre-infection physical exercise decreases mortality and stimulates neurogenesis in bacterial meningitis

**DOI:** 10.1186/1742-2094-9-168

**Published:** 2012-07-10

**Authors:** David Liebetanz, Joachim Gerber, Christina Schiffner, Sandra Schütze, Florian Klinker, Hubertus Jarry, Roland Nau, Simone C Tauber

**Affiliations:** 1 Department of Clinical Neurophysiology, Georg-August-University, Robert-Koch-Strasse 40, 37075 Göttingen, Germany; 2 Department of Neurology, Diana Hospital, Dahlenburger Strasse 2a, 29549 Bad Bevensen, Germany; 3 Department of Neurology, RWTH University Hospital, Pauwelsstrasse 30, 52074 Aachen, Germany; 4 Department of Neuropathology, Georg-August-University, Robert-Koch-Strasse 40, 37075 Göttingen, Germany; 5 Department of Geriatrics, Ev. Krankenhaus Weende, An der Lutter 24, 37075 Göttingen, Germany

**Keywords:** Exercise, Survival, Mortality, Neurogenesis, *Streptococcus pneumoniae*, Bacterial meningitis

## Abstract

Physical exercise has been shown to increase neurogenesis, to decrease neuronal injury and to improve memory in animal models of stroke and head trauma. Therefore, we investigated the effect of voluntary wheel running on survival, neuronal damage and cell proliferation in a mouse model of pneumococcal meningitis. Mice were housed in cages equipped with voluntary running wheels or in standard cages before induction of bacterial meningitis by a subarachnoid injection of a *Streptococcus pneumoniae* type 3 strain. 24 hours later antibiotic treatment was initiated with ceftriaxone (100 mg/kg twice daily). Experiments were terminated either 30 hours or 4 days (short-term) or 7 weeks (long-term) after infection, and the survival time, inflammatory cytokines and corticosterone levels, neurogenesis in the dentate gyrus of the hippocampal formation and the cognitive function were evaluated in surviving mice. Survival time was significantly increased in running mice compared to control animals (*p* = 0.0087 in short-term and *p* = 0.016 in long-term experiments, log-rank test). At the end of the long-term experiment, mortality was lower in trained than in sedentary animals (*p* = 0.031, Fisher’s Exact test). Hippocampal neurogenesis – assessed by the density of doublecortin-, TUC-4- and BrdU + NeuN-colabeled cells - was significantly increased in running mice in comparison to the sedentary group after meningitis. However, Morris water maze performance of both groups 6 weeks after bacterial meningitis did not reveal differences in learning ability. In conclusion, physical exercise prior to infection increased survival in a mouse model of bacterial meningitis and stimulated neurogenesis in the dentate gyrus of the hippocampal formation.

## Introduction

Mortality in bacterial meningitis is high, amounting to approximately 25% in adults, and survivors frequently suffer from long-term sequelae, particularly from learning and memory deficits [1,2]. Neuronal cell damage is evident in the hippocampal formation, the neocortex, the basal ganglia, and the brain stem of victims of bacterial meningitis [3,4]. Additionally, axonal injury in the white matter has been reported in animal meningitis models and in human bacterial meningitis [5,6].

While acute diseases of the brain regularly cause cell damage and neuronal death, endogenous mechanisms of cell proliferation in neuronal precursor cells and stimulation of neurogenesis in response to brain injury have been observed in several diseases of the central nervous system (CNS), for example, in cerebral ischemia [7,8], in head trauma [9], after epileptic seizures [10], in hypoxic-ischemic encephalopathy [11] and in septic-metastatic encephalitis [12]. Similarly, hippocampal neurogenesis was also increased after bacterial meningitis in experimental models and in a human autopsy study [13-15]. Stimulation of neurogenesis, however, was found not only in response to damage of the CNS but also under conditions of physical exercise. In healthy mice, enriched environment and exercise increased proliferation of progenitor cells in the hippocampal formation [16,17]. Furthermore, exercise improved both neurogenesis and learning in young and aged mice, thereby providing evidence for a beneficial effect of motor activity on brain function [17,18].

In animal models of brain diseases such as stroke and traumatic injury, physical exercise was also able to mediate neuroprotection and to reduce memory deficits observed regularly in these diseases. In this study, we investigated if physical exercise by voluntary wheel running prior to induction of bacterial meningitis is able to alleviate the course of the disease, to strengthen the power of resistance against bacterial infection and to increase neurogenesis.

## Material and methods

### Mouse model and experimental design

For all experiments, male C57BL/6 mice (weight 18 to 22 g, age 6 to 8 weeks) were used. To prove whether running alone exerts stimulating effects on hippocampal neurogenesis, mice were housed separately in either standard laboratory cages (24 × 18 × 16 cm) (n = 10) or cages equipped with voluntary running wheels (n = 10) for a duration of 4 weeks. These mice were not infected but were sacrificed after 4 weeks. To investigate whether running exerts positive effects on the course of meningitis, mice that were later infected, were either housed separately in standard laboratory cages or in cages equipped with voluntary running wheels (n = 54). Duration and velocity of voluntary wheel running was recorded by a computer system (Runningwheel Systems, TSE Systems, Bad Homburg, Germany and Boenig & Kallenbach oHG, Dortmund, Germany). Control animals were kept under the same conditions but without running wheels (n = 54). Four (n = 15) and two (n = 39) weeks after voluntary wheel running, bacterial meningitis was induced in all animals: mice were intraperitoneally anesthetized with 100 mg/kg body weight ketamine and 10 mg/kg xylazine before injection of 10 μL of 0.9% NaCl containing 10^4^ colony-forming units (CFU) of a *Streptococcus pneumoniae* type 3 strain into the subarachnoid space through the right frontolateral skull. All animals developed clinical signs of infection within 24 hours. Clinical score [14] and weight were evaluated twice daily during the acute phase of meningitis (5 days) and thereafter once weekly. Starting 24 hours after infection, mice received subcutaneous antibiotic treatment twice daily with 100 mg/kg body weight ceftriaxone (Roche, Grenzach-Wyhlen, Germany) either for 3 days until the end of experiments 4 days after infection (short-term group), or for 5 days in the long-term group with termination of the experiments 7 weeks after infection. Mice that were sacrificed 30 hours after infection (n = 30) for analysis of inflammatory parameters and corticosterone plasma concentrations did not receive antibiotic treatment. In these animals blood samples were taken immediately before infection and 30 hours after infection at the time of sacrifice by retro-orbital puncture. Additionally, mice participating in the long-term experiment received a total dose of 250 mg/kg body weight bromodeoxyuridin (BrdU) intraperitoneally in five doses of 50 mg/kg body weight BrdU every 12 hours from day 2 after infection. During the acute phase of the disease, 11 mice of the short-term group with antibiotic treatment (36 to 86 hours after infection) and 16 animals of the long-term group (24 to 130 hours after infection) died. The short-term experiments were terminated 4 days (108 hours) after infection. In the long-term experiments, mice were evaluated weekly for motor function by the tight rope test as described previously [14]. Six weeks after infection, Morris water maze was performed (further details in the next paragraph). At the end of the experiments, all surviving animals of both groups were sacrificed by intraperitoneal injection of an anesthetic agent, and cerebellum, spleen and blood samples were taken for the analysis of bacterial titers or cytokine levels, and the brains fixed in formalin for further immunohistochemical analysis. All animal experiments were approved by the Animal Care Committee of the University Hospital of Göttingen and by the District Government of Braunschweig, Lower Saxony, Germany as well as by the Animal Care Committee of the University Hospital of Aachen and by the District Government in Recklinghausen, North Rhine-Westphalia, Germany.

### Morris water maze

The water maze task was performed in a pool, painted white (diameter 100 cm, height 40 cm) containing water maintained at a temperature of 22 °C. A transparent platform 10 cm in diameter was located 1 cm beneath the water surface. The pool was surrounded by several visual cues that were external to the maze. A video camera mounted to the ceiling above the pool was linked to a computer system (Video tracking system Any-maze, Stoelting, IL, USA) and recorded the swim tracks and time required to escape from the water. Mice were trained to find the hidden platform within less than 90 seconds (30 trials over 5 days), and the latency, swim speed and distance to find the submerged platform were evaluated (spatial acquisition).

### Assessment of bacterial titers, cytokine and corticosterone levels

Bacterial titers were evaluated 30 hours after infection in tissue homogenates of spleen and cerebellum and in blood samples by plating 10-fold dilutions on blood agar plates and incubating for 24 hours at 37 °C and 5% CO_2_ (detection limit 10^2^ CFU/mL in tissue homogenates and 10^3^ CFU/mL in blood samples). TNF alpha and IL-6 were chosen as representatives of inflammatory mediators in bacterial meningitis. TNF alpha levels were determined using antibody pairs from BioLegend (Biozol, Munich, Germany), and a DuoSet ELISA Development Kit (R&D Systems, Wiesbaden, Germany) was used for the measurement of IL-6. The color reaction was measured at 450 nm on a microplate reader (Bio-Rad, Munich, Germany). The measurement of corticosterone levels was carried out by ELISA (BO1263, Beckman Coulter, Krefeld, Germany). The lowest analytical detectable level of corticosterone that could be distinguished from the zero calibrator was < 1 ng/mL.

### Immunohistochemistry

Formalin-fixed and paraffin-embedded 3 μm coronal brain sections (according to coordinates from bregma, 1.46 to 1.77 mm [19]) were used for the immunohistochemical analysis. After deparaffinization, sections were pretreated with 5 × 3 minutes of microwaving in citric acid buffer, 10 mmol/l, pH 6.0. After blocking with 10% FCS/PBS, primary antibodies were applied at the concentrations indicated below and permitted to bind overnight at 4 °C (TUC-4) or for 90 minutes at room temperature (profilerating cell nuclear antibody (PCNA), doublecortin (Dcx). For quantification of cell proliferation, a monoclonal mouse anti-PCNA antibody was used (1:200; Chemicon, Temecula, CA, USA). Then sections were incubated with appropriate biotinylated secondary antibodies (Amersham, Little Chalfont, Buckinghamshire, UK) followed by an avidin-peroxidase treatment (Sigma-Aldrich, St. Louis, MO, USA). Expression of TUC-4 was determined by binding of 1:1000 diluted polyclonal rabbit anti-TUC-4 antibody (Chemicon, Temecula, CA, USA), and detection was performed with the alkaline phosphatase/anti-alkaline phosphatase (APAAP) method and visualized by newfuchsin. Dcx was detected by a rabbit polyclonal anti-Dcx antibody (1:1000; Abcam, Cambridge, UK). Sections were incubated with appropriate biotinylated secondary antibodies (Amersham, Buckinghamshire, UK) followed by treatment with avidin-peroxidase (Sigma-Aldrich, St. Louis, MO, USA). Diaminobenzidine (DAB) was used as the chromogenic substrate. Control sections were incubated with isotype control antibodies or without primary antibody as negative control. To analyze how many BrdU-immunoreactive cells had differentiated into mature neurons, double immunofluorescence labeling of BrdU with the neuron-specific nuclear antigen (NeuN) was performed as follows: after pretreatment by microwaving in citric acid buffer, slices were blocked with 10% FCS/PBS for 30 minutes. Sections were then incubated with 1:500 diluted monoclonal rat anti-BrdU antibody (Abcam, Cambridge, UK) overnight at room temperature. Sections were then incubated for one hour with 1:500 anti-rat Alexa Fluor 594 (Invitrogen, Eugene, OR, USA). This was followed by incubation with monoclonal mouse anti-NeuN (1:1000, Millipore, Schwalbach, Germany) for three hours at room temperature. Finally, slices were incubated with anti-mouse Alexa Fluor 488 (Invitrogen, Eugene, OR, USA) for one hour.

### *In situ* tailing

Apoptotic cells were identified by *in situ* tailing and morphologic criteria (condensed, shrunken nuclei and narrow cytoplasms). Deparaffinized and hydrated 2-mm sections were treated with 50 mg/ml proteinase K (Sigma, Deisenhofen, Germany) for 15 minutes at 37 °C in a reaction mixture containing 10 ml of 5 × tailing buffer, 1 ml digoxigenin DNA labeling mix, 2 ml cobalt chloride, 12.5 U terminal transferase and the necessary amount of distilled water to give a volume of 50 ml. After washing, the sections were incubated with 10% FCS for 15 minutes at room temperature and then washed again. A solution of alkaline phosphatase-labeled anti-digoxigenin antibody in 10% FCS (1:250) was placed on the sections for 60 minutes at 37 °C. The color reaction (black) was developed with 4-nitroblue-tetrazolium-chloride/5-bromine-4-chloride-3-indolyl-phosphate. Sections were counterstained with nuclear fast red-aluminiumhydroxide (all reagents from Roche, Mannheim, Germany).

### Quantification of immunoreactive cells

The sections were examined blinded using a 2 × objective up to a 60 × objective. Only immunoreactive cells within the granule cell layer and subgranular zone of the dentate gyrus were counted. An Analysis Software Imaging System (microscope BX51; Olympus, Hamburg, Germany; software AnalySIS® 3.2; Soft Imaging System GmbH, Münster, Germany) was used to measure the area of the dentate granule cell layer. The density of immunolabeled cells was evaluated in three coronal sections from each mouse and expressed as the number of marked cells per mm^2^ of the area measured. The density of BrdU-labeled cells and the proportion of proliferating cells differentiating into mature neurons were determined by evaluation of the percentage of BrdU-labeled proliferative cells that co-expressed the marker NeuN 7 weeks after meningitis.

### Statistical analysis

Survival time was expressed in hours and evaluated by generating a Kaplan-Meier plot that was statistically analyzed using the log-rank test. Overall mortality at the end of the experiment was compared by Fisher`s exact test. Running distance and maximum velocity were expressed as the mean and standard error of the mean (SEM). Bacterial titers were converted into log CFU/ml and compared by unpaired *t*-test. Immunohistochemical data and cytokine measurements were expressed as median and interquartile range. For statistical comparison, the nonparametric Mann–Whitney *U*-test was used. Values of the water maze were expressed as median and interquartile range and statistically analyzed by nonparametric repeated measures analysis of variance (ANOVA). A value of *p* < 0.05 was considered statistically significant. GraphPad Prism (version 5.0) or Statistical Analysis Software (SAS) (version 9.1) were used for statistical calculations. Graphs were generated using GraphPad Prism.

## Results

### Voluntary wheel-running activity four weeks prior to infection

**Figure 1:**
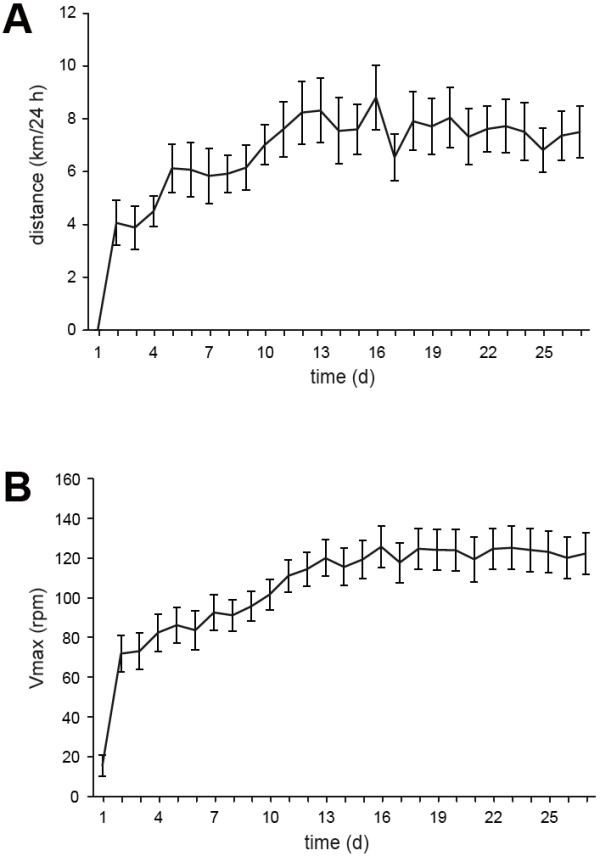
**Running activity over 4 weeks.** The mean daily running distance of the mice during voluntary wheel running exercise (n = 15) was recorded in km per day. (**A**) During the first 2 weeks, mice increased their wheel running capacity, which was accompanied by an improvement in maximum wheel running velocity (Vmax) during this episode. (**B**) Both performance parameters remained stable during the following plateau phase (mean ± standard error of the mean, SEM).

The time the mice spent running daily, the running distance and the maximum velocity increased each day during the first days until a plateau was reached after the second week that was maintained until the end of the study (Figure 1AB). This course of the running activity indicated that the animals performed an intensive cardiopulmonary and musculoskeletal exercise after an initial training phase. The average running distance accumulated to 179.7 km during 27 days. The mean daily running distance was 6.7 ± 0.35 km/day.

### Motor performance

The motor performance assessed by the tight rope test was severely impaired during acute bacterial meningitis with the highest score (that is, the poorest motor performance) at 36 hours after infection. Motor function had recovered in survivors by day 4. There was no significant difference between running mice and controls in motor performance during meningitis (*P* = 0.21). The weight of mice suffering from bacterial meningitis decreased during acute disease, with the maximum weight loss corresponding to the poorest motor performance at 36 hours after infection. Thereafter, weight normalized back to the pre-infection level within 6 days. These observations confirmed the severity of the infection.

### Inflammatory reaction during bacterial meningitis

There were no significant differences in clinical score or weight between runners and sedentary mice during the acute phase of meningitis (clinical score of runners 0.87 ± 0.35 vs. 0.87 ± 0.52 30 hours after infection, *P* = 1.0, unpaired *t*-test; weight before infection in runners 24.2 ± 1.2 g vs. 23.8 ± 1.0 in controls, *P* = 0.36, unpaired *t*-test; weight 24 h after infection 21.3 ± 0.8 vs. 21.7 ± 1.1, *P* = 0.27, unpaired *t*-test). Comparison of bacterial load in cerebellum, spleen and blood 30 hours after infection showed a tendency towards a lower bacterial burden in runners, but this difference was not statistically significant. Bacterial counts in blood samples were 4.45 ± 0.9 log CFU/mL in runners vs. 4.72 ± 0.88 log CFU/mL in controls (*P* = 0.44, unpaired *t*-test) and in spleen homogenates they were 3.64 ± 0.65 log CFU/mL in runners vs. 3.84 ± 0.95 log CFU/mL in controls (*P* = 0.514, unpaired *t*-test); bacterial titers in cerebellar homogenates were 6.16 ± 0.46 log CFU/mL in runners and 6.14 ± 0.56 log CFU/mL in control animals (*P* = 0.952, unpaired *t*-test).

Determination of IL-6 and TNF alpha in blood samples and homogenates of the cerebellum 30 hours after infection revealed that the protein expression of IL-6 was much higher in both compartments in comparison to TNF alpha. Levels of TNF alpha in the blood were below the 15 pg/ml limit of quantification. Overall there was a tendency to lower levels of both cytokines in cerebellum and IL-6 in the blood of runners compared to control animals. For IL-6 in cerebellum and blood, the medians (25th/75th percentiles) in pg/ml were 3,495 (2,767/4,599) vs. 3,975 (1,916/4,625) and 862 (659/1025) vs. 943 (815/1,150) in runners and controls respectively, and for TNF alpha in cerebellum they were 72 (44/96) vs. 78 (65/118).

As expected, comparison of corticosterone plasma levels before and 30 hours after infection revealed a distinct increase after infection in both runners and sedentary mice (*P* < 0.0001, Mann–Whitney *U*-test, n = 15). There was a tendency towards lower corticosterone levels in running animals in comparison to controls prior to infection but this difference was not statistically significant (*P* = 0.14, Mann–Whitney *U*-test, n = 15). Before infection, the medians (25th/75th percentiles) for corticosterone in ng/ml were 6.03 (1/14.9) vs. 11.06 (1.4/16.8) ng/ml respectively in runners and controls, and 30 hours after infection were 564.6 (513.3/588.1) vs. 532.3 (475.3/565.8).

### Running increased neurogenesis in the dentate gyrus of the hippocampal formation in the absence of meningitis

#### *Four weeks of running did not change proliferation but the density of dentate granule cells expressing early neuronal markers*

PCNA is expressed during the S-phase of mitosis and is used as a marker for cellular proliferation and DNA synthesis. In this study, the density of PCNA-immunoreactive proliferating cells showed no difference between runners and control mice after 4 weeks of running (*P* = 0.91, Mann–Whitney *U*-test, n = 20); the median (25th/75th percentile) was 55.9 (43.2/86.5)/mm^2^ for runners and 72.5 (50.3/80.9)/mm^2^ for controls.

TUC-4 (also TOAD-64) is a membrane-associated marker, which is transiently expressed in the cytoplasm and processes of young but not adult neuronal cells [20,21]. Dcx is a protein required for normal migration of neurons into the cerebral cortex and is transiently expressed in newly formed neuroblasts [22]. The expression of TUC-4 and Dcx was used to identify the population of newborn cells developing into the neuronal cell lineage. There was a tendency to more dentate granule cells expressing TUC-4 in running mice compared to the sedentary group but this difference was not statistically significant (*P* = 0.212, Mann–Whitney *U*-test, n = 20). The median (25th/75th percentile) was 67.1 (9.5/116.3)/mm^2^ for runners and 29.6 (15.2/42.4)/mm^2^ for controls. The density of Dcx-expressing granule cells was significantly higher in running animals compared to sedentary mice 4 weeks after running (*P* = 0.0005, Mann–Whitney *U*-test, n = 20). The median (25th/75th percentile) was 206.7 (164.4/265.3)/mm^2^ for runners and 102.1 (54.5/162.6)/mm^2^ for controls.

### Exercise increased survival time and decreased mortality in bacterial meningitis

#### *Four weeks of running prior to meningitis increased survival time in short-term experiments*

Five animals out of fifteen in the running group and six out of fifteen in the sedentary group (approximately 33% in total) died in the acute phase of bacterial meningitis. The survival time after induction of infection, however, was significantly longer in the running group in comparison to that of the sedentary group. The median survival time (25th/75th percentile) was 76 (38/86) hours in the running group and 27 (25/35) hours in the control group (*P* = 0.0087, log-rank test). Since 4 weeks of running exerted significant effects, the running time was reduced to 2 weeks to assess whether short-term exercise also affected survival parameters.

#### *Two weeks of running prior to meningitis decreased mortality in long-term experiments*

**Figure 2:**
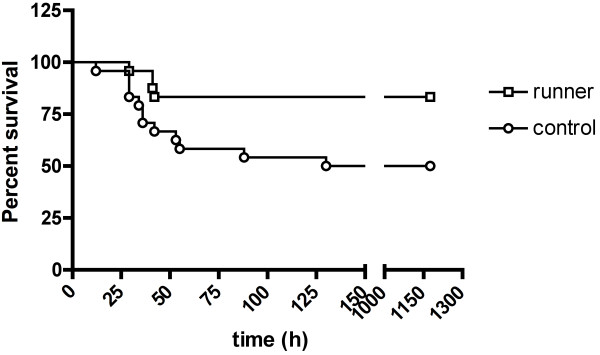
**Comparison of survival during the course of meningitis with or without preceding running activity.** Kaplan-Meier survival curves illustrating the course of *Streptococcus pneumoniae* meningitis, revealing increased survival in running mice (open squares) in comparison to sedentary controls (open circles) (*P* = 0.016, log-rank test, n = 48).

The course of the Kaplan-Meier survival curves of trained and sedentary animals was significantly different, demonstrating prolonged survival in trained animals (*P* = 0.016, log-rank test, Figure 2). Sixteen out of forty-eight animals died during the course of bacterial meningitis (33%). From these 16 animals, 12 belonged to the sedentary group and 4 were runners. In this series of experiments, exercise prior to meningitis led to a statistically significant decrease in mortality after meningitis (*P* = 0.031, Fisher’s exact test).

### Proliferation and synthesis of young neurons in the dentate gyrus four days after bacterial meningitis (short-term experiments)

#### *No evidence for exercise-induced proliferation four days after meningitis*

The density of PCNA-immunoreactive proliferating cells did not differ between runners and control animals 4 days after infection (*P* = 0.90, Mann–Whitney *U*-test, n = 19), the median (25th/75th percentile) was 126.3 (55.6/180)/mm^2^ for runners and 119 (72.3/166.5)/mm^2^ for controls.

#### *Exercise stimulated expression of TUC-4 and Dcx in the dentate gyrus after meningitis*

**Figure 3:**
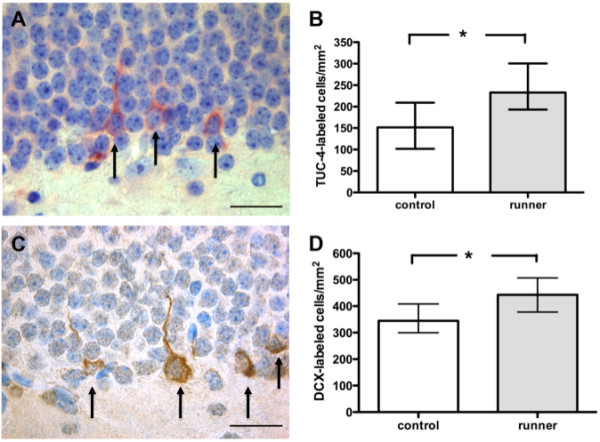
**Increased hippocampal neurogenesis in running mice four days after bacterial meningitis.** Four days after infection, the density of TUC-4- (**A**, **B**) as well as doublecortin-expressing (**C**, **D**) young neurons was significantly increased in the granule cell layer of the dentate gyrus after voluntary wheel running in comparison to sedentary mice. (**B**) and (**D**) represent the median and interquartile range of the densities of immunoreactive cells (example in **A** und **C**)/mm^2^ dentate gyrus, *P* = 0.012 and *P* = 0.015, respectively, Mann–Whitney *U*-test, scale bar = 50 μm).

The density of dentate granule cells expressing TUC-4 was higher in running mice compared to the sedentary group (*P* = 0.012, Mann–Whitney *U*-test, n = 19, Figure 3A and B). Similarly, Dcx-expressing cells were observed more frequently in the granule layer of the dentate gyrus in running animals compared to sedentary mice (*P* = 0.015, Mann–Whitney *U*-test, n = 19, Figure 3C and D).

#### *No evidence of axonal damage or increased neuronal apoptosis after running*

Axonal injury, assessed by evaluation of APP staining as a marker of acute axonal damage, was not detected in either group. Furthermore, analysis of apoptotic neurons by *in-situ* tailing did not reveal differences in the rate of apoptosis between runners and controls; the median (25th/75th percentile) was 0 (0/1.6)/mm^2^ in controls and 0 (0/1.9)/mm^2^ in runners (*P* = 0.88, Mann–Whitney *U*-test, n = 19).

### Changes in hippocampal neurogenesis seven weeks after bacterial meningitis (long-term experiments)

#### *Exercise did not increase the number of proliferating cells in the dentate gyrus*

The density of BrdU-immunoreactive cells in the granule cell layer was lower in running mice in comparison to sedentary animals (*P* = 0.01, Mann–Whitney *U*-test, n = 32). The median (25th/75th percentile) was 29.8 (23.6/47.2)/mm^2^ in runners and 72.7 (51.8/96.4)/mm^2^ in controls.

#### *No change in the number of Dcx-expressing cells in the dentate gyrus seven weeks after meningitis*

In contrast to observations 4 days after meningitis, the density of Dcx-immunoreactive cells did not differ significantly between runners and sedentary control animals 7 weeks after infection; the median (25th/75th percentile) was 180.5 (137.1/207.8)/mm^2^ vs. 128.5 (82.58/189.4)/mm^2^ in runners and controls respectively (*P* = 0.18, Mann–Whitney *U*-test, n = 32).

#### *Higher proportion of BrdU/NeuN co-labeled cells after exercise*

**Figure 4:**
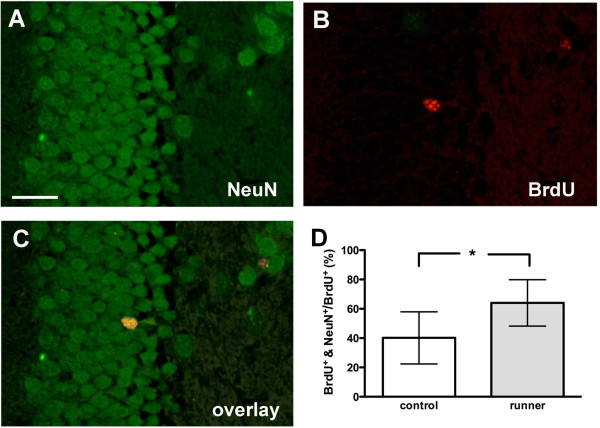
**Increased hippocampal neurogenesis in runners seven weeks after bacterial meningitis.** Detection of NeuN (**A**) and BrdU (**B**) by double-label fluorescent immunohistochemistry. Merger of both markers revealed a newly formed neuron 7 weeks after bacterial meningitis (**C**). There was a higher percentage of co-labeled BrdU + NeuN + neurons in running mice in comparison to sedentary controls (**D**) (median and interquartile range, *P* = 0.018, Mann–Whitney *U*-test, scale bar = 50 μm). BrdU: Bromodeoxyuridin; NeuN: Neuron-specific nuclear antigen.

To determine the differentiation of proliferating cells into adult neurons in the subgranular zone of the dentate gyrus 7 weeks after bacterial meningitis with or without preceding exercise, the proportion of BrdU-immunoreactive cells that co-expressed the neuron-specific marker NeuN was evaluated. The percentage of proliferating cells having differentiated into mature neurons was significantly higher in running mice with meningitis in comparison to sedentary mice with meningitis (*P* = 0.018, Mann–Whitney *U*-test, n = 32, Figure 4).

### Morris water maze task

Six weeks after initiation of the experiment, the Morris water maze task was carried out. Exercise prior to bacterial meningitis did not improve performance in this task in comparison to sedentary mice. The groups did not perform differently in the Morris water maze with respect to latency to reach the hidden platform (*P* = 0.4594, repeated measures ANOVA), swim velocity (*P* = 0.9453, repeated measures ANOVA) and distance traveled to locate the platform (*P* = 0.3446, repeated measures ANOVA, n = 32, data not shown).

## Discussion

Exercise and voluntary wheel running have not only been shown to increase neurogenesis [17] but also to improve memory and learning tasks [18] and exert beneficial effects on diseases of the CNS, such as stroke, by facilitating motor recovery in rats [23]. Frequent long-term sequelae after bacterial meningitis are cognitive deficits as a consequence of hippocampal damage in the course of this infectious disease. Conversely, hippocampal neurogenesis has been shown to increase after meningitis in both rodents and humans [13-15], but the effects of CNS inflammation on neurogenesis remain controversial [24,25].

The increase in neurogenesis after brain injury possibly reflects an endogenous potential of cell replacement. Mice exposed to an enriched environment after recovery from bacterial meningitis, as a rehabilitative approach, did not show additive elevation of the meningitis-induced increase of hippocampal neurogenesis, suggesting a limited capacity of the brain to further increase the already elevated neurogenesis after bacterial meningitis. Accordingly, learning performance was not better in mice exposed to an enriched environment after meningitis compared to animals with meningitis who received no further stimuli [26]. The present study aimed to investigate whether exercise prior to infection could alleviate the course of disease and increase neurogenesis, as well as improve spatial learning performance, thereby attenuating long-term sequelae after meningitis.

In this study, mortality due to *Streptococcus pneumoniae* meningitis was significantly decreased in mice that had been exercised by means of running wheels prior to infection, compared to animals that were kept under standard conditions. It can be hypothesized that improved cardiorespiratory capacity achieved by regular exercise facilitated survival and that an altered immune response towards a less pro-inflammatory direction was also responsible for decreased mortality. Indeed, the decrease of mortality was accompanied by a tendency towards lower bacterial burden as well as towards lower pro-inflammatory cytokine levels of IL-6 and TNF alpha and lower corticosterone levels in runners compared to sedentary mice. Overall, the extent of the inflammatory and stress response to bacterial meningitis was decreased by physical exercise. This observation is in accordance with other studies showing a decreased release of TNF alpha in response to lipopolysaccharide challenge in physically active animals [27], and adjuvant therapy with TNF alpha converting enzyme inhibitor-prolonged survival in rats with *Streptococcus pneumoniae* meningitis [28]. Similar to this mouse model of meningitis, cortisol levels were significantly increased in the cerebrospinal fluid of patients with pneumococcal meningitis [29], and running appeared to have beneficial effects by reducing pre-infection corticosterone levels.

Since proliferative activity showed no difference between running and sedentary mice 4 days after infection, we propose that running prior to infection was unable to further increase the cell proliferation in the dentate gyrus stimulated also by the infection, but favored the differentiation of new-born cells into the neuronal lineage. Comparison of BrdU-labeled cells 7 weeks after meningitis revealed a lower amount of proliferating cells in running mice. One cannot differentiate whether this difference is due to fewer proliferating cells in the acute phase of meningitis (BrdU was applied on day 3 to 5 after induction of meningitis) or whether more initially labeled cells died or went into apoptosis by the end of the experiments 7 weeks after meningitis.

The number of TUC-4 and Dcx-expressing young neurons in the dentate gyrus was significantly increased in running mice 4 days after infection, suggesting a stimulating effect of physical exercise on neurogenesis in addition to the stimulating effects of meningitis alone. Seven weeks after meningitis, the number of Dcx-expressing cells no longer differed between the groups, but the percentage of initially proliferating BrdU-labeled cells that differentiated into adult neurons was significantly higher in running mice in comparison to sedentary controls. Running probably exerted its stimulating effects on neurogenesis by modulating the inflammatory response and reducing corticosterone levels, as the latter has already been shown in healthy mice [30]. However, this modulation may not be the only reason for the beneficial effects of running. Other parameters such as the modulation of growth factors may also contribute to the increase of neurogenesis after meningitis and exercise. Promotion of growth factor pathways by running resulted in both stimulation of hippocampal neurogenesis and in functional improvement of memory tasks [31]. In a rat model of traumatic brain injury, physical exercise 6 weeks before head trauma resulted in a better adaptation to oxidative challenge and prevented against ROS (reactive oxygen species)-mediated inhibition of the Na+, K + −ATPase [32].

The increase of hippocampal neurogenesis in this study was not accompanied by improved spatial learning: no difference in Morris water maze performance was observed between runners and non-runners 6 weeks after meningitis. If there were possible positive effects of running on cognitive performance, these effects were most probably erased by the damage caused by meningitis, and running prior to meningitis did not overcome these post-inflammatory sequelae. Whether learning and hippocampal neurogenesis positively correlate is still a matter of debate. There are studies postulating a correlation between neurogenesis and cognitive performance [33-37] while other studies imply partially contradictory results [13,26,38-41]. Furthermore, there are studies in which adult neurogenesis was virtually completely suppressed by radiation [42] or genetic manipulation [43] and in which learning and memory performance of the rodents remained unimpaired [44-47]. These divergent results may be in part a consequence of technical differences and varying time points of evaluation [48]. The detection of alterations in spatial learning in the Morris water maze is a challenge because even healthy rodents without any manipulation perform differently in this task depending on species differences, genetic background, high intraindividual variation and motivation; all of which lowers the chance of detecting significant differences [49].

In conclusion, voluntary wheel running prior to the induction of the infection prolonged the survival time and decreased the mortality of bacterial meningitis, attenuated the inflammatory response and, furthermore, increased the differentiation of proliferating cells into adult neurons in the dentate gyrus suggesting a beneficial and protective effect of running. Physical exercise increased the ability of the host organism to survive *Streptococcus pneumoniae* meningitis with the aid of antibiotic therapy, but the neuroregenerative effect of this mechanism with respect to neuropsychological outcome appeared small under the conditions of the present study.

## Abbreviations

ANOVA, analysis of variance; APAAP, alkaline phosphatase/anti-alkaline phosphatase; BrdU, bromodeoxyuridin; CFU, colony-forming units; CNS, central nervous system; DAB, diaminobenzidine; Dcx, doublecortin; ELISA, enzyme-linked immunosorbent assay; FCS, fetal calf serum; IL, interleukin; IST, in-situ tailing; NeuN, neuron-specific nuclear antigen; PBS, phosphate-buffered saline; PCNA, proliferating cell nuclear antigen; ROS, reactive oxygen species; SEM, standard error of the mean; TNF, tumor necrosis factor.

## Competing interests

The authors declare that they have no competing interests.

## Authors’ contributions

DL made substantial contributions to the conception and design of the study, carried out part of the animal experiments and contributed to the interpretation and discussion of the data. JG contributed to the design of the experiments, carried out part of the animal experiments and helped with the discussion of the data. CS accomplished all neuropsychological tests and performed the immunohistochemistry. FK carried out part of the animal experiments. SS carried out the animal experiments in the course of the revision of the manuscript and measured the cytokine levels. HJ measured the corticosterone levels in the course of the revision of the manuscript. RN helped with the interpretation and discussion of the data and with drafting the manuscript. SCT supervised all experimental procedures, carried out part of the animal experiments, supervised and evaluated the neuropsychological study procedures, analyzed the immunohistochemical data, performed all statistical analysis and wrote and revised the manuscript. All authors read and approved the final version of the manuscript.
